# Differential expression of the *Slc4* bicarbonate transporter family in murine corneal endothelium and cell culture

**Published:** 2013-05-24

**Authors:** William Shei, Jun Liu, Hla M. Htoon, Tin Aung, Eranga N. Vithana

**Affiliations:** 1 Singapore Eye Research Institute, Singapore; 2 Department of Ophthalmology, National University Health System & National University of Singapore, Singapore; 3 Singapore National Eye Centre, Singapore; 4 Neuroscience and Behavioral Disorders (NBD) Program, Duke-NUS Graduate Medical School, Singapore

## Abstract

**Purpose:**

To characterize the relative expression levels of all the solute carrier 4 (*Slc4*) transporter family members (*Slc4a1–Slc4a11*) in murine corneal endothelium using real-time quantitative (qPCR), to identify further important members besides *Slc4a11* and *Slc4a4*, and to explore how close to the baseline levels the gene expressions remain after cells have been subjected to expansion and culture.

**Methods:**

Descemet’s membrane-endothelial layers of 8–10-week-old C57BL6 mice were stripped from corneas and used for both primary cell culture and direct RNA extraction. Total RNA (from uncultured cells as well as cultured cells at passages 2 and 7) was reverse transcribed, and the cDNA was used for real time qPCR using specific primers for all the *Slc4* family members. The geNorm method was applied to determine the most stable housekeeping genes and normalization factor, which was calculated from multiple housekeeping genes for more accurate and robust quantification.

**Results:**

qPCR analyses revealed that all *Slc4* bicarbonate transporter family members were expressed in mouse corneal endothelium. *Slc4a11* showed the highest expression, which was approximately three times higher than that of *Slc4a4* (3.4±0.3; p=0.004). All *Slc4* genes were also expressed in cultured cells, and interestingly, the expression of *Slc4a11* in cultured cells was significantly reduced by approximately 20-fold (0.05±0.001; p=0.000001) in early passage and by approximately sevenfold (0.14±0.002; p=0.000002) in late passage cells.

**Conclusions:**

Given the known involvement of *SLC4A4* and *SLC4A11* in corneal dystrophies, we speculate that the other two highly expressed genes in the uncultured corneal endothelium, *SLC4A2* and *SLC4A7*, are worthy of being considered as potential candidate genes for corneal endothelial diseases. Moreover, as cell culture can affect expression levels of *Slc4* genes, caution and careful design of experiments are necessary when undertaking studies of *Slc4*-mediated ion transport in cultured cells.

## Introduction

Bicarbonate ions have been implicated to play a central role in human corneal endothelial ion pump to maintain corneal transparency [1]. A substantial proportion of cellular HCO_3_^–^ transport is mediated by proteins belonging to the solute carrier 4 (SLC4) family. The SLC4 family of transporters consists mainly of three functional groups: 1) electroneutral Na^+^-independent Cl^–^/HCO_3_^-^exchangers, 2) electrogenic or electroneutral Na^+^:HCO_3_^–^ cotransporters, and 3) electroneutral Na^+^-driven Cl^–^/HCO_3_^–^ exchangers [2,3]. SLC4A11, the most divergent member of this family, has been described to function as an electrogenic Na^+^-coupled borate co-transporter and as an electrogenic Na^+^/OH^–^ co-transporter in the absence of borate [4].

Several members of the *SLC4* gene family have been linked to ocular and corneal diseases in humans. Mutations in *SLC4A4* cause proximal renal tubular acidosis as well as ocular anomalies, such as glaucoma [5], cataracts, and band keratopathy [6]. Mice lacking sodium bicarbonate cotransporter (NBC3; *Slc4a7*) develop blindness and auditory impairment due to the degeneration of sensory receptors in the eye and inner ear [7]. Moreover, *SLC4A11* was identified to be responsible for three corneal endothelial dystrophies, recessive congenital hereditary endothelial dystrophy (CHED2) [8], late onset Fuchs endothelial dystrophy (FECD) [9], and corneal dystrophy with perceptive deafness (Harboyan syndrome) [10].

Despite the importance of HCO_3_^–^ transport in the normal functioning of the cornea and the involvement of some of its members in corneal dystrophies, the entire *SLC4* gene family has not been systematically characterized in the cornea. The expression of *SLC4A4,* which encodes the electrogenic sodium bicarbonate cotransporter NBC1, has been localized to the basolateral membrane of corneal endothelial cells [11-13]. Gene expression of *SLC4A11* has been shown in the human corneal endothelium via SAGE and microarray [14] as well as reverse transcription PCR [15]. Gene expression information is lacking for the other members of the *SLC4* family.

Therefore, the purpose of this study was to characterize the expression levels of the entire *Slc4* family of genes relative to those of *Slc4a4* and *Slc4a11* in the mouse corneal endothelium to identify further *SLC4* members that can serve as candidate genes for analysis in corneal dystrophies. The expressional alterations that occur for *Slc4* genes due to cell culturing procedures involving both early and late subcultures were also explored.

## Methods

### Animal experimentation

C57BL6 wild-type mice were ordered from the animal holding unit of the National University of Singapore and housed and bred in the Singhealth Experimental Medicine Center until a sufficient number for the study was obtained. Approval was sought from the SingHealth International Animal Care and Use Committee (IACUC), and all procedures were in accordance with the Association for Research in Vision and Ophthalmology resolution for the use of animals in research.

### Primer design

PCR primers were designed for all members of *Slc4* gene family (*Slc4a1* to *Slc4a11*) as well as housekeeping genes (HKGs; glyceraldehyde-3-phosphate dehydrogenase[*Gapdh*], 18s ribosomal RNA [*18s rRNA*], beta-actin [*Actb*], hypoxanthine phosphoribosyltransferase 1 [*Hprt1*]) and cell identifying marker genes (Aquaporin 1 [*Aqp1*], Zona occludens 1 [*Zo 1*], Collagen group VIII a2 [*Col8a2*], Collagen group I a1 [*Col1a1*]). The primers for the target genes were designed based on the mouse mRNA sequence using Primer 3 primers design software [16]. Forward and reverse primers were designed to be located on separate exons (with a large intron in between) to ensure that the template used would be cDNA rather than genomic DNA. Each primer sequence was queried against the mouse DNA NCBI database, using BLAST to ensure that primer sequences were specific for the target mRNA transcript. The primers were also designed such that they were in a region that detects all known splice variants of the corresponding transcript. The primers were synthesized by AIT Biotech (Singapore). The optimized primer sequences used in this study are listed in the Table 1.

**Table 1:** The sequences of the primers used in the study

Gene Name	Orientation	Sequence	Product size
*Slc4a1*	Forward	CTCCTTCCTCATCTCCCTCA	104
	Reverse	TCATCACAACAGGGGCATAA	
*Slc4a2*	Forward	ATGTGGCCTCACTGTCCTTC	124
	Reverse	ATCTGCTCGACCACCTGATG	
*Slc4a3*	Forward	ATTCCCATCTCCATCCTGGT	110
	Reverse	CGCTTATGAGGGGAAGTCAC	
*Slc4a4*	Forward	TCCCTTCATTGCCTTTGTTC	151
	Reverse	CAAGGTGGCGATAGCTCTTC	
*Slc4a5*	Forward	TGAACACAACCACGGTCAAT	126
	Reverse	CGTAGCTCAGGCACTCCTTC	
*Slc4a7*	Forward	CGCATAGAGCCTCCAAAAAG	113
	Reverse	GCATGGTGATCATCCTCCTT	
*Slc4a8*	Forward	GGGCAGCAGTACCATGAGAT	126
	Reverse	GTCCAGGAACTCGTCAATCC	
*Slc4a9*	Forward	CCTTGGCCCACATAGACAGT	119
	Reverse	TGTAAGGACGAACACCACCA	
*Slc4a10*	Forward	TTCAAGACCAGCCGCTATTT	109
	Reverse	GGATCCCAATGGCATAGTCA	
*Slc4a11*	Forward	CTGTGAGGTTCGCTTTGTCA	138
	Reverse	GTGCGAGTCTTCAGGAGCTT	
*Gapdh*	Forward	GCTACACTGAGGACCAGGTTG	150
	Reverse	TGCTCTTAAAAGTCAGGTTTCC	
*18s rRNA*	Forward	AAACGGCTACCACATCCAAG	112
	Reverse	CAATTACAGGGCCTCGAAAG	
*Actb*	Forward	CTAAGGCCAACCGTGAAAAG	104
	Reverse	ACCAGAGGCATACAGGGACA	
*Hprt1*	Forward	CAAACTTTGCTTTCCCTGGT	100
	Reverse	CTGGCCTGTATCCAACACTTC	
*Aqp1*	Forward	CTACACTGGCTGCGGTATCA	143
	Reverse	GGGCCAGGATGAAGTCATAG	
*Zo 1*	Forward	GGCTTAGAGGAAGGTGATCAAA	100
	Reverse	CTTTAGGGAGGTCAAGGAGGA	
*Col8a2*	Forward	AGGGTCCAGTAGGGGCTAAA	100
	Reverse	CCCTTAGGTCCTGGTTTTCC	
*Col1a1*	Forward	CTTCACCTACAGCACCCTTGTG	85
	Reverse	CTTGGTGGTTTTGTATTCGATGAC	

### Isolation of mouse corneal endothelial cells

Adult 8–10-week-old C57BL6 wild-type mice (n=5 for the primary cell culture and n=10 for direct RNA extraction) were sacrificed with an overdose of sodium pentobarbital. Eyes were enucleated, and the globes were rinsed with sterile 1X PBS to remove traces of blood and other material. The corneas were then dissected from the globe and laid endothelial side up in a sterile Petri dish. Descemet’s membrane (DM)–endothelial layers were stripped under a dissecting microscope and incubated overnight to stabilize the cells in Opti-MEM I^®^ (Invitrogen, Carlsbad, CA) medium. Endothelial cells were separated from the DM by 2 mg/ml collagenase A (Sigma, St. Louis, MO) treatment at 37 °C for 2–3 h in minimum essential media (MEM, Invitrogen) supplemented with 15% fetal bovine serum (FBS) and 20 μg/ml gentamicin. After detachment from DMs, the mouse corneal endothelial cells (MCECs) were subjected to direct RNA extraction or culture.

### Mouse corneal endothelial cell culture

MCECs were washed with Dulbecco’s Modified Eagle’s Medium (DMEM, Invitrogen) supplemented with 0.1 mg/ml gentamicin and 1.25 μg/ml amphotericin B before culturing in MEM medium supplemented with 15% FBS and 20 μg/ml gentamicin in a humidified atmosphere with 5% CO_2_ at 37 °C. The cells were passaged on reaching 80% confluence. The MCECs at passage 2 and passage 7 were used for subsequent RNA extraction. The entire experiment was done three times independently to obtain three separate batches of cells at passages 2 and 7.

### RNA isolation and reverse transcription PCR

Total RNA from mouse uncultured corneal endothelial cells as well as cultured cells at passages 2 and 7 were isolated by the TRIZOL™ (Invitrogen) method following the manufacturer’s protocol with a few modifications. Briefly, the stripped Descemet’s membranes or the harvested cells were homogenized and lysed in the TRIZOL™ reagent (Invitrogen). Then 0.2 ml chloroform was added to each 1 ml of TRIZOL™ reagent, and centrifuged at 10,000 ×g for 20 min at 4˚C. The aqueous layer was mixed with 0.5 ml isopropanol and incubated overnight. The reaction was centrifuged at 10,000 ×g for 20 min at 4˚C, isopropanol was removed and mixed with 1 ml of cold 75% ethanol. The RNA wash with ethanol was done twice by centrifugation at 7500 ×g for 4 min at 4˚C and the resulting pellet was dissolved in RNase free water. Genomic DNA was removed by digestion with DNase I (AmpGrade; Invitrogen-Gibco) according to manufacturer’s protocol. Genomic DNA was removed by digestion with DNase I (AmpGrade; Invitrogen-Gibco). The dissolved RNA sample was measured on a spectrophotometer (Nanodrop 2000; Thermo Fischer Scientific, Waltham, MA) to determine the concentration and quality before proceeding to convert to cDNA. One microgram of total RNA was reverse transcribed with random hexamers by using SuperScript III™ first-strand synthesis system for reverse transcription (RT)-PCR (Invitrogen). A traditional PCR amplification was performed in a 50-μl reaction volume using GoTaq^®^ DNA Polymerase (Promega, Madison, WI), an equal amount of first-strand cDNA template, and the optimized primers in a thermal cycler GeneAmp^®^ PCR System 9700 (Applied Biosystems, Carlsbad, CA). We applied the following PCR parameters: 95 °C for 3 min, followed by 40 cycles of 95 °C for 30 s, 58 °C for 30 s, and 73 °C for 90 s. The resulting PCR products were separated by 2% agarose gel electrophoresis. The gel was then viewed under ultraviolet illumination, and the images were taken by a Hamamatsu image detection system (Hamamatsu, Japan).

### Immunofluorescence staining

Cells were seeded on coverslips until approximately 80% confluent. The cells were fixed in 4% paraformaldehyde for 30 min at 4 °C, incubated separately with primary anti-Na^+^K^+^ ATPase (200 µg/ml; Santa Cruz Biotechnology, Santa Cruz, CA) and anti-Zo-1antibodies (5 µg/ml; Life tech, Carlsbad, CA) at recommended dilutions (1:100 for Na^+^K^+^ ATPase and 1:50 for Zo-1) for 2 h at room temperature, washed three times with 1X PBS, and then incubated with Alexa Fluor^®^ 488-conjugated secondary antibody (Invitrogen) for 1 h. Nuclear DNA was visualized by staining with DAPI. The cells were mounted on mounting medium (VECTORSHEILD, Vector Laboratories, Burlingame, CA) and then examined using a fluorescent microscope with an ApoTome attachment (Axio Imager Z1; Zeiss, Stuttgart, Germany).

### Selection of the most stable housekeeping gene

The most stable HKG was selected by using the geNorm™ VBA applet for Microsoft Excel version 3.5 (Biogazelle, Gent, Belgium) according to the manufacturer’s protocol. Briefly, the expression data matrix with raw data of relative quantities of each HKG was loaded to calculate the M values, which measure gene expression stability, and the gene expression normalization factor for each sample based on the geometric mean. The detailed underlying principles and calculations were described by Vandesompele et al. [17].

### Real-time quantitative PCR

qPCR with SYBR^®^ Green I dye for detection was performed in a 10-μl mixture containing 5 μl of SYBR^®^ green PCR master mix (Applied Bisosystems), 500 nM of each primer, and 1 μl of cDNA template, using the LightCycler^®^ 480 (Roche, Basel, Switzerland). The threshold cycles (Ct) were calculated using the LightCycler^®^ software version 1.5 (Roche). The cycling parameters used for amplifications of targets were as follows: initial denaturation for 10 min at 95 °C, followed by 45 cycles of 30 s denaturation at 95 °C, 30 s annealing at 58 °C, and 45 s extension at 72 °C. The expression levels of *Slc4* bicarbonate transporters were normalized against that of the most stable HKG *Hprt1*, as shown by the geNorm™ analysis. Relative quantification using the comparative Ct method was used to analyze the data output. Values were expressed as the fold change over corresponding values for the control by the 2^–ΔΔCt^ method [18].

### Statistical analysis

All the data used for statistical analyses were obtained from three independent experiments. Differences between the groups was determined by the two-tailed Student *t* test using spreadsheet software (Excel 5.0; Microsoft, Redmond, WA), with significance determined at p<0.05.

## Results

### **Analysis of *Slc4* gene expression in murine uncultured corneal endothelial cells** Determining amplification efficiency and quality of the primers

In order for the ΔΔCt method to be valid, the amplification efficiencies for both the tested gene and the HKG must be optimally equal to 2 (100% efficiency). Therefore, we validated the amplification efficiencies of all primers of the *Slc4* family as well as the HKGs by real-time qPCR, using the mouse kidney cDNA as the standard template. Mouse kidney cDNA was used for this purpose since all *Slc4* genes were shown to be expressed in mouse kidney tissue [19] and sufficient quantities of RNA could be extracted from kidney tissue. Six serial dilutions of the template were subject to real-time qPCR to investigate the amplification efficiency of the primers. All the primers showed similar amplification efficiencies in the serially diluted samples of mouse kidney cDNA (Table 2). To ensure a single product was obtained without primer dimers, we also performed a melting curve analysis to verify that a single specific melting peak was observed for each primer pair.

**Table 2:** The amplification efficiencies for all reference and target genes

Gene	Slope	Efficiency (E)
*Slc4a1*	-3.55	1.91
*Slc4a2*	-3.51	1.92
*Slc4a3*	-3.32	2
*Slc4a4*	-3.58	1.9
*Slc4a5*	-3.17	2.07
*Slc4a7*	-3.52	1.92
*Slc4a8*	-3.48	1.94
*Slc4a9*	-3.56	1.91
*Slc4a10*	-3.17	2.06
*Slc4a11*	-3.13	2.08
*Actb*	-3.3	2
*Hprt1*	-3.15	2.08
*Gapdh*	-3.08	2.11
*18s rRNA*	-3.23	2.04

The efficiency is described as time of PCR product increase per cycle by the formula E=10^–1/slope^. Consistent, high amplification efficiencies (95 - 105%) were achieved in all cases.

### Preliminary semiquantitative analysis

**Figure 1:**
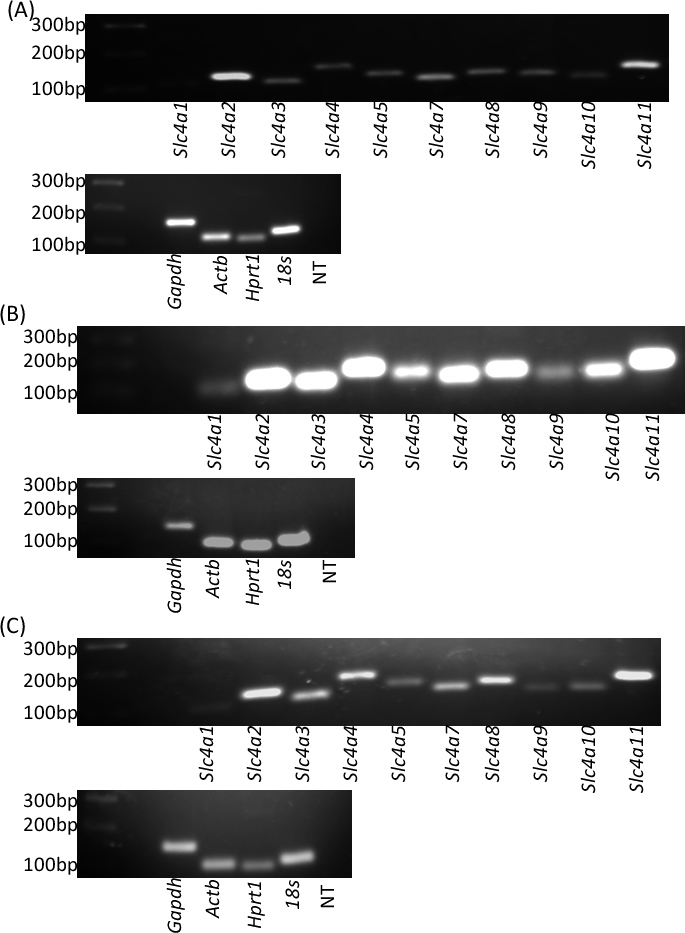
Analysis of *Slc4* gene expression by reverse transcription polymerase chain reactions (RT–PCR). Template cDNA samples were generated from mouse primary corneal endothelium (**A**) and cultured mouse corneal endothelial cells at passage 2 (**B**) and at passage 7 (**C**). A 100 base pair (bp) marker is shown in the first lane from the left. The primers used to generate the PCR products (Table 1) are indicated below each lane. Four housekeeping genes (*Gapdh*, *Actb*, *Hprt1*, and *18s rRNA*) were used as amplification (positive) controls. Each set of reactions (per gene) included a no-template (NT) negative control; these samples were pooled and the combined sample was subject to electrophoresis.

The standard PCR with Taq DNA polymerase using the mouse corneal endothelial cDNA template and primers that were selected specifically to target the mRNA of the *Slc4* genes revealed a single RT-PCR product of the predicted size for a given mRNA template for each primer pair. This indicated that there was no contamination by genomic DNA. The four HKGs (*Gapdh*, *Actb*, *Hprt1*, *18s rRNA*) were used as amplification (positive) and normalizing controls. This analysis revealed that all *Slc4* genes were expressed in murine corneal endothelium (Figure 1A). It also indicated *Slc4a11* and *Slc4a2* genes had the highest expression levels, while *Slc4a1* and *Slc4a9* appeared to have the lowest expression levels in uncultured corneal endothelium. However, RT-PCR is semiquantitative and not as sensitive (or always accurate) as real-time qPCR for the assessment of relative gene expression.

### *Slc4a11*, the most highly expressed gene in murine uncultured corneal endothelium

**Figure 2:**
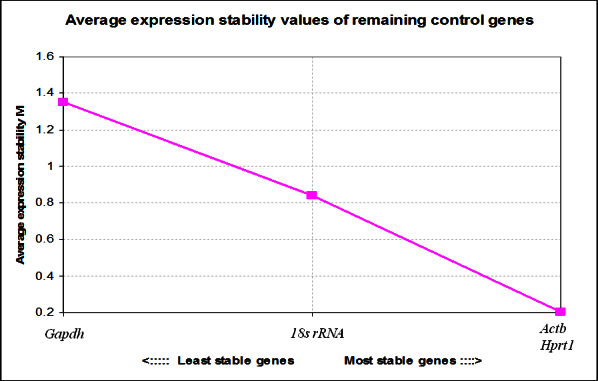
GeNorm™ analysis. The most stable reference genes were selected from the four most commonly used housekeeping genes (*Gapdh*, *Actb*, *18s rRNA*, and *Hprt1*) using the geNorm™ software. They were systematically compared with each other, and the resulting average expression stability plot was used to rank each housekeeping gene in order of expression stability. The genes with the lower average expression level stability (M) values are considered to have more stable expression levels.

Further analysis by real-time qPCR using the most stable HKG, *Hprt1* (Figure 2) revealed that among the *Slc4* genes, *Slc4a11* had the highest expression level while *Slc4a1* showed the lowest (Table 3). The order of expression level was therefore *Slc4a11* followed by *Slc4a2, Slc4a4, Slc4a7, Slc4a3, Slc4a10, Slc4a5, Slc4a9, Slc4a8,* and *Slc4a1.* The expression of *Slc4a2* was approximately half that of *Slc4a11* (0.55±0.03; p=0.007), while the expression of *Slc4a1* was 300 times less than *Slc4a11* (0.0026±0.0006; p=0.003). When comparing the expression levels of the two clinically important genes known for corneal diseases (i.e., *Slc4a11* and *Slc4a4*), the expression of *Slc4a11* was approximately three times greater than that of *Slc4a4* (3.4±0.3; p=0.004).

**Table 3:** Relative normalized mRNA expression levels of *Slc4* gene family in mouse corneal endothelium

Gene name	Normalized expression (relative to *Slc4a11*)	P value	Normalized expression (relative to *Slc4a4*)	P value
*Slc4a1*	0.0026±0.0006	0.00297*	0.0088±0.0022	0.00091*
*Slc4a2*	0.5548±0.0293	0.00701*	1.8856±0.1022	0.00098*
*Slc4a3*	0.1632±0.0272	0.00219*	0.5548±0.0926	0.00253*
*Slc4a4*	0.2942±0.016	0.00416*	1.0000±0.0542	-
*Slc4a5*	0.0341±0.0019	0.00309*	0.1158±0.0064	0.00097*
*Slc4a7*	0.2432±0.0222	0.00308*	0.8265±0.0755	0.00507*
*Slc4a8*	0.0051±0.0005	0.00300*	0.0175±0.0014	0.00096*
*Slc4a9*	0.0100±0.0015	0.00297*	0.0338±0.0049	0.00087*
*Slc4a10*	0.0509±0.0102	0.00266*	0.1731±0.0347	0.00019*
*Slc4a11*	1.0000±0.0953	-	3.3987±0.3241	0.00416*

Data shown are the expression levels of a given gene relative to that of *Slc4a11* or *Slc4a4*. P values indicate the significance between the normalized expression of given gene and normalized expression of the calibrator gene (*Slc4a11* or *Slc4a4*) from three independent experiments and in this experimental context indicate the reproducibility of relative expression data. The asterisks indicate statistical significance at p < 0.05.

### **Investigation of *Slc4* family expression in cultured mouse corneal endothelial cells** Morphology of mouse corneal endothelial cells

**Figure 3:**
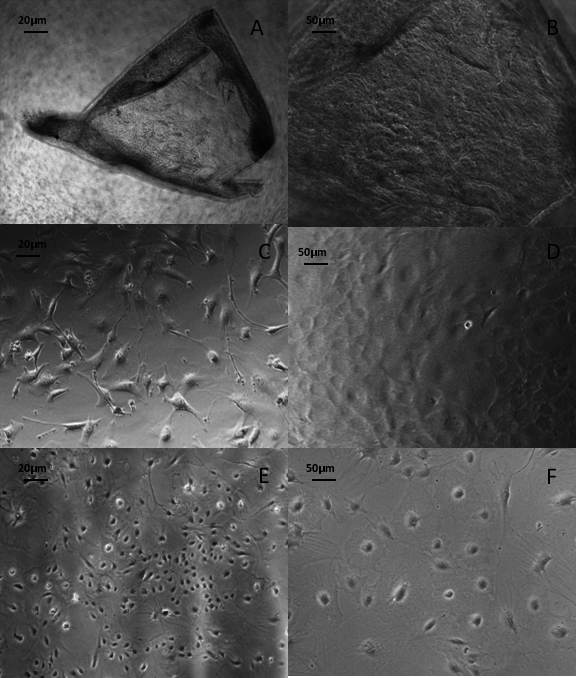
Establishment of mouse corneal endothelial cell (MCEC) cultures. The phase contrast micrographs show the Descemet’s membrane–endothelial layers stripped from corneas of wild-type mice (**A**, scale bar = 20 µm; **B**, scale bar = 50 µm). The morphology of cultured MCECs as observed at early passage 2 (**C**, scale bar = 20 µm; **D**, scale bar = 50 µm) and at late passage 7 (**E**, scale bar = 20 µm; **F**, scale bar = 50 µm).

The MCECs initially showed stellate morphology at low densities, and upon confluence they became polygonal in shape, characteristic of endothelial cells (Figure 3C,D). Cultures were split (i.e., passaged) upon reaching 80% confluence, and passaging was performed until the seventh passage for this study. In the latter passages, some cells were more elongated in appearance, while some appeared larger in size with more prominent nuclei (Figure 3E,F).

### Cultured mouse corneal endothelial cells expressed corneal endothelial markers

**Figure 4:**
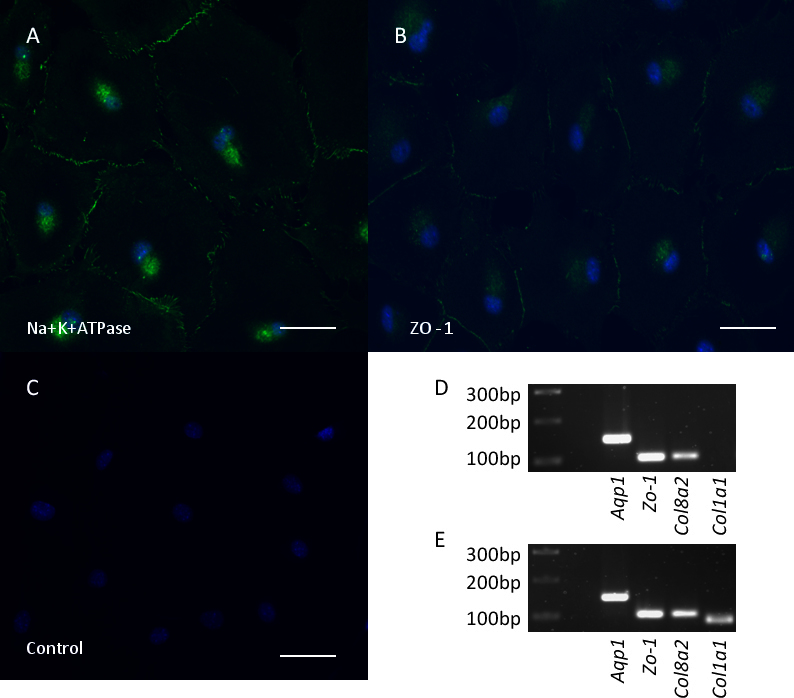
Characterization of mouse corneal endothelial cells (MCECs). MCECs in passage 2 were immunostained with the antibodies for Na+K+ ATPase (**A**), Zo-1 (**B**), Isotype-matched IgG1 negative control (**C**) and visualized by fluorescent microscopy. Scale bar: 50 μm. Reverse transcription PCR was also carried out to see mRNA expression of *Aqp1*, *Zo-1*,*Col8a2*, *Col1a1* in cultured passage 2 MCECs (**D**) and passage 7 MCECs (**E**). Size markers are shown in the first lane from the left. Note that the fibroblast marker *Col1a1* was not expressed in passage 2 cells but was expressed in passage 7 cells.

To characterize the MCECs, immunofluorescent analyses were performed on the cultured passage 2 MCECs with antibodies for Na^+^K^+^ATPase and Zo-1. Na^+^K^+^ATPase is involved in physiologic maintenance of corneal thickness by the corneal endothelium [20,21], while Zo-1 is a selective semipermeable tight junction-associated protein in the corneal endothelium [22,23]. The cultured MCECs expressed both Na^+^K^+^ ATPase and Zo-1 (Figure 4A,B). We further characterized the cells by examining the expressions of several genes normally present in corneal endothelial cells (*Aqp1, Zo-1, Col8a2*) as well as the fibroblastic marker gene *Col1a*1 by RT- PCR (Figure 4D,E). MCECs at both early and late passages expressed the endothelial markers *Aqp1*, *Zo-1,* and *Col8a2*. The fibroblast cell marker *Col1a1* was not expressed by early passage 2 cells but was expressed by late passage 7 cells. RT-PCR also revealed the expression of all *Slc4* genes in the cultured MCECs (Figure 1B,C).

### Alteration in expression of *Slc4* genes in cultured mouse corneal endothelial cells

**Figure 5:**
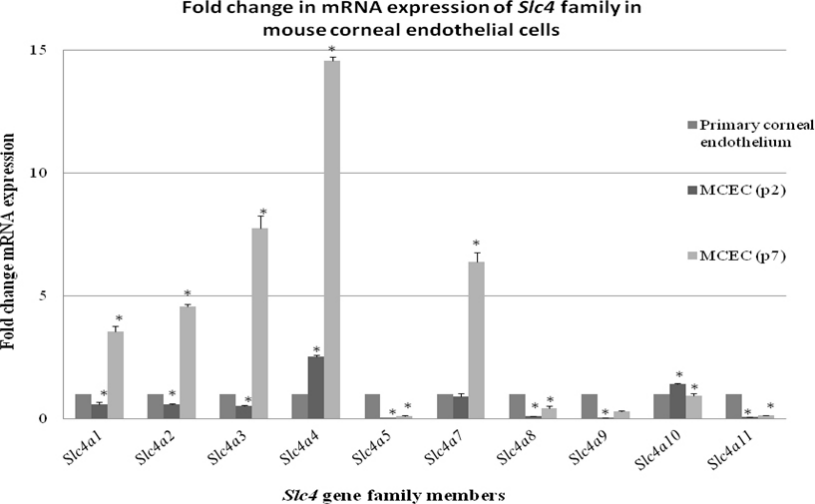
*Slc4* gene expression in cultured mouse corneal endothelial cells. The expression of *Slc4* genes was compared between cultured cells (passage 2 and 7) and the primary corneal endothelium, which served as the calibrator sample. Gene expression was normalized using a normalization factor, calculated from expression data of three housekeeping genes (*Hprt1*, *Actb* and *18s rRNA*). Results shown are the mean fold change ± standard deviation in mRNA expression relative to the calibrator sample (set as 1) calculated from three independent experiments. The asterisks indicate statistical significance at p<0.05.

Real-time qPCR using the normalization factor calculated from *Hprt1*, *Actb,* and *18s rRNA* (Table 4) indicated that the expressions of all the *Slc4* transporters, except *Slc4a4* and *Slc4a10,* were downregulated in early passage 2 (Table 5). However, in late passage 7, *Slc4a1, Slc4a2, Slc4a3, Slc4a4,* and *Slc4a7* were upregulated. The expression of *Slc4a11* was significantly reduced by 20-fold (0.05±0.001; p=0.000001) in early passage but only by sevenfold (0.14±0.002; p=0.000002) in late passage. Interestingly, the expression of another clinically important gene, *Slc4a4,* was significantly upregulated by approximately 2.5-fold (2.52±0.07; p=0.0007) in passage 2 and 14-fold (14.57±0.16; p=0.00005) in late passage 7 (Figure 5).

**Table 4:** Calculation of normalization factors for most stable reference genes

Cell source	*18s rRNA*	*Actb*	*Hprt1*	Norm. Factor
Cultured MCEC (passage 2)	1.00	1.00	1.00	1.00
Cultured MCEC (passage 7)	0.14	0.27	0.32	0.2313
Primary corneal endothelium	0.76	0.34	0.37	0.4564
M <1.5	1.323	0.973	0.963	

The geometric means of the most stable reference genes were calculated to obtain the normalization factor. MCEC, mouse corneal endothelial cells; *18s rRNA*, 18s ribosomal RNA; *Actb,* β actin; *Hprt1*, Hypoxanthine phosphoribosyltransferase 1; Norm factor, normalization factor.

**Table 5:** Alteration in mRNA expressions of *Slc4* family genes in cultured (passage 2 and 7) mouse corneal endothelial cells compared to the primary corneal endothelium

Gene name	Fold change in mRNA expression of MCECs (p2)	P value	Fold change in mRNA expression of MCECs (p7)	P value	Significance between p2 and p7
*Slc4a1*	0.5697±0.0902	0.01433*	3.5308±0.2449	0.00311*	0.00091*
*Slc4a2*	0.5939±0.0130	0.00034*	4.5631±0.0813	0.00017*	0.00010*
*Slc4a3*	0.5046±0.0293	0.00116*	7.7543±0.5002	0.00182*	0.00140*
*Slc4a4*	2.5286±0.0733	0.00077*	14.5707±0.1649	0.00005*	0.00002*
*Slc4a5*	0.0166±0.0028	0.00000*	0.1081±0.0180	0.00014*	0.00914*
*Slc4a7*	0.9033±0.1080	0.26121	6.3643±0.4105	0.00195*	0.00102*
*Slc4a8*	0.0847±0.0184	0.00013*	0.4104±0.1147	0.01238*	0.02797*
*Slc4a9*	0.0401±0.0093	0.00003*	0.3035±0.0080	0.00004*	0.00001*
*Slc4a10*	1.3931±0.0404	0.00350*	0.9428±0.0791	0.33701	0.00246*
*Slc4a11*	0.0525±0.0014	0.00000*	0.1441±0.0020	0.00000*	0.00002*

Data shown are the relative fold change in gene expression in cultured cells over primary corneal endothelium that served as the calibrator sample. Mean ± standard deviation values obtained from three independent culturing experiments are shown. The P values in last column indicate the significance between p2 and p7 fold change values from three independent experiments. The asterisks indicate statistical significance at p < 0.05.

## Discussion

Previous studies have investigated *SLC4* family expression in other types of tissue [24] but not in ocular tissues. We found that all members of the *Slc4* bicarbonate transporter family are expressed in MCECs, and some genes, such as *Slc4a4, Slc4a2,* and *Slc4a7,* are more highly expressed than others. Although *SLC4A4* and *Slc4a7* have been reported to be associated with ocular conditions in humans [5,6] and mice [7], respectively, a documented link between *SLC4A2* and an eye disease is currently lacking. These genes can, therefore, serve as potential candidate genes to be analyzed for corneal endothelial diseases, such as FECD. We observed a similar result for humans, with these genes being among the highest expressed in corneal endothelial cells (unpublished data). *Slc4a11* showed the highest expression in uncultured CECs, indicating that it plays a pivotal role in transporting solutes in the corneal endothelium, although we did not establish a functional correlation with its expression. The exact function of SLC4A11 in the corneal endothelium is unknown. However, the congenital corneal opacity seen in recessive CHED cases as well as the severe morphological alterations displayed by *Slc4a11* mutant mice attests to the importance of SLC4A11 in maintaining normal corneal endothelial function [25].

The SLC4 family, except sodium-coupled borate cotransporter (SLC4A11), is functionally divided into three main groups [26,27]: anion exchangers (AEs), sodium bicarbonate cotransporters (NBCs), and sodium-driven Cl^–^/HCO_3_^–^ exchangers (NDCBE). We found that the expression of genes encoding the two NBCs (*Slc4a4* and *Slc4a7*) to be most highly expressed after *Slc4a11*, indicating that the NBCs are the main bicarbonate transporters in corneal endothelium. Our data indicated that for NBCs, NBCe1 (*Slc4a4)* is the primary member in the corneal endothelium and NBCn1 (*Slc4a7)* is secondary (Table 6). Similarly for AEs, AE2 (*Slc4a2*) appears to be primary and AE3 (*Slc4a3*) secondary. For the NDCBE family of proteins, AE1 (*Slc4a1)* is primary and NDCBE *(Slc4a8)* secondary. The redundancy seen with this family of genes is perhaps indicative of the important role that these genes play in ion transport within the corneal endothelium. It will also be interesting to explore the compensatory role played by the secondary gene in the event of a defect, i.e., mutation, involving the primary member of each class. Understanding the hierarchy of expression of bicarbonate transporters within the same functional group has also opened up the interesting possibility of compensatory therapeutics.

**Table 6:** Proposed hierarchy for *Slc4* family members within a given functional group

Functional group	Primary	Secondary
Na bicarbonate cotransporters (NBCs)	NBCe1 (*Slc4a4)*	NBCn1 (*Slc4a7)*
Anion exchangers (AEs)	AE2 (*Slc4a2*)	AE3 (*Slc4a3*)
Na-driven Cl^-^/HCO_3_^-^ exchangers (NDCBEs)	AE1 (*Slc4a1)*	NDCBE *(Slc4a8)*

The genes were categorized according to their relative levels of expression in mouse corneal endothelium.

In this study, we found that *Slc4* gene expression was significantly altered during cell culture. Interestingly, the expression of *Slc4a11* gene was reduced by sevenfold in late passages while that of another clinically important gene, *Slc4a4*, was significantly up-regulated by approximately 2.5-fold in early passages and 14-fold in late passages. The morphology of endothelial cells was also less “endothelial like” in late passages. The environmental conditions of the cultured cells, for example the high salt content of the culture media, could be a key factor for the altered expression of solute transporters seen in cultured cells compared to native tissue. Another possible explanation for the observed alterations in gene expression is the epithelial/endothelial-to-mesenchymal transition (E/EnMT) during which endothelial cells lose endothelial markers and obtain mesenchymal markers, as suggested by the expression of *Col1a1* in late passage cells in our study (see Figure 4E). In vitro studies have shown that endothelial cells can undergo EMT, which is speculated to depend on transforming growth factor ß1 [28,29]. The drastically altered expression levels of the *Slc4* genes coincident with an altered cellular morphology indicate that further study should be undertaken to explore the possible link between *SLC4* gene expression and EMT. More importantly, the effects of cell culture on the expression levels of *Slc4* genes highlight the need for caution and the careful design of experiments with adequate controls when studying ion transport activity of these proteins in cultured cells.

The main limiting factor in this study is that *Slc4* gene expression was only tested at the RNA level and not at the protein level. Ideally, the expression levels should have been confirmed by using specific antibodies to the various *Slc4* genes through western analysis of protein extracts. The lack of commercially available antibodies for murine Slc4 members that are sensitive or specific for immunoblot analysis precluded further confirmation of our results at the protein level.

To the best of our knowledge, this is the first study to investigate transcript expression levels of the entire *Slc4* bicarbonate transporter family in mouse corneal endothelial cells. We could establish expression profiles for each member in primary corneal endothelium as well as quantify the expressional alterations that occur for *Slc4* genes due to our cell culturing procedure that involved both early and late subcultures. Identification and characterization of more genes causative of corneal endothelial diseases would further our understanding of pathologic mechanisms underlying this group of disorders and may lead to novel ways to treat these conditions.
